# About Pocket Surgical Cases

**Published:** 1864-03

**Authors:** E. Andrews

**Affiliations:** Professor of Surgery in Chicago Medical College


THE
### CHICAGO MEDICAL EXAMINER.

N. S. DAVIS, M.D., Editor.

VOL. V. MARCH, 1864. NO. 3.

### Original Contributions.

ARTICLE IX.

### ABOUT POCKET SURGICAL CASES.

By E. ANDREWS, M.D., Professor of Surgery in Chicago Medical College.

The object of a case of pocket surgical instruments is, that the practitioner may always have with him a small supply of such instruments as he most frequently uses, and a few others which, though not so often required, yet are occasionally called for with such urgency and suddenness as to admit of no delay. The size of the case should be as small as possible, and the material of it such as to be durable, handsome, and capable of excluding moisture.

Unfortunately, most of the cases which are sold, are combined by the makers without sufficient consultation with surgeons; hence the material of the case is generally morocco, which soils, wears out, and always admits dampness, and the interior of it is lumbered up with an excessive variety of forceps and scissors to the exclusion of better things. Several passable cases, however, have been produced, among which, for a small one, Parker's case is one of the best, and for a fuller one, Reed's case. Both, however, are made of morocco, and like others of that sort, soon lose their comliness, and do not perfectly protect the instruments.

On these accounts, I resolved to have a new case constructed, with a new selection of instruments, such as experience showed to be most convenient. I therefore made drawings and descriptions of a case such as I desired, and requested TIEMAN & Co., of New York, to manufacture and keep them among their stock, which they readily undertook to do.

The material of the case is polished hard rubber, perfectly impervious to water, and having almost the beauty of jet. It is, therefore, extremely durable, and will retain its beauty and polish for indefinite years. In fact, the case will be unimpaired when the instruments are worn out by long use.

It opens on a strong hinge, in two equal valves, exposing all the instruments to view at once, ready to be taken up for use. When closed it is about $5\frac{1}{2}$ inches in length, $2\frac{3}{4}$ inches in breadth, and $\frac{7}{8}$ of an inch in thickness. The ends and all the edges are rounded to prevent wearing the pocket, and the whole appearance is much more beautiful and solid than that of leather and morocco cases.

The selection of instruments is as follows:—

| | |
|---|---|
| 1 Scalpel,<br>1 Probe pointed bistoury, | both in one handle. |
| 1 Tenotome,<br>1 Sharp-pointed bistoury, | " " " |
| 1 Gum Lancet,<br>1 Tenaculum, | " " " |

1 Pair of Scissors.
1 Rat-toothed or bulldog forceps.
1 Dressing Forceps.
1 Silver catheter in 4 pieces, which can be combined into a male, or female catheter, as desired.
1 Porte caustique.
1 Silver probe.
1 Silver director.
1 Pocket case saw.
1 Silk, ivory, or wooden card for holding ligature.
6 Suture needles.

I find by experience that this combination is extremely convenient for the following reasons:—The first six instruments, of course, are found in every pocket case having any pretension to completeness, and need no remarks. Of the scissors there is only one pair, and that of the small straight variety. This form is by far the best for general purposes, and the insertion of two or three varieties of scissors into one pocket case is a very inconvenient waste of room. With the straight scissors one can do almost everything required, including even the operations for entropium and strabismus. For forceps, I prefer the small rat-toothed variety, without any lock, but others choose the bulldog form. Either can be put in, but it is not best to fill up the case with both. The dressing forceps, so called, is of the kind resembling polypus forceps. It serves a very valuable purpose, not only in handling dressings, but also in extracting fragments of necrosed bone from deep fistulas. It is also an excellent bullet forceps for pistol balls which have not penetrated very deeply, and has the merit of being able, in cases of impacted balls, to bite out and bring away a small chip of lead, thus settling the question of whether the body seized is really the bullet, or only the edge of a bone. While in the army, I found this to be a very important quality in some difficult diagnoses. It also serves as a very excellent polypus forceps for the removal of polypus nasi.

The compound silver catheter needs no explanation. It is capable of being turned into a male or female instrument as required. It seems not generally known among physicians that with a clean silver catheter and a scalpel or lancet, the operation of tapping can be performed with as much neatness and perfection as with the best trochar.

The pocket case saw is a most important instrument, and yet very seldom seen. The form heretofore made is that of a delicate saw set in a tortoise shell handle, and shutting up like a scalpel. This has three faults. 1st. The blade can be no longer than the handle. 2d. The junction of the saw with the handle is liable to break under hard usage, and thus totally disable the instrument. 3d. The combined thickness of the saw and of the handle causes it to occupy too much room in the case. For these reasons I devised a saw of which the handle and blade are all in one piece of steel, and thin enough to lie in the case beneath another instrument, thus practically taking up no room. It has thus strength and compactness. With this saw a femur or a humerus can be sawn off without difficulty. Hence in an emergency, by means of this pocket case alone, the surgeon need not hesitate to undertake the amputation of an arm or a thigh. With the scalpel incisions may be made on the circular plan, or, if preferred, flaps can be cut from without inward, as advised by Dr. Prince, of Jacksonville. Practically, however, the pocket case saw comes oftenest into use in fractures for cutting and modifying splints to fit the patient. For this purpose I find it an absolute necessity. It is also available as a metacarpal saw in minor amputations.

The ivory card, which may be made also of wood, shell, or horn, is by far the best implement for holding ligature silk. It keeps it perfectly straight, smooth, and unentangled. It is notched at the extremities, and the silk wound on lengthwise, and the whole held in a loop like any other instrument.

The suture needles, instead of being stuck in a piece of paper or cloth, are neatly deposited in a small box in one valve of the case, and covered with their inside lid. All these instruments are held in position by loops of elastic ribbon, which keep everything securely in place, even if the case is opened and held bottom upwards. The whole inside is lined with velvet, and a fly pocket for containing adhesive strap is attached between the two valves. The price of the whole is about $25, and it may be ordered either of Tieman & Co., of New York, or of Bliss & Sharp, Chicago.

